# Interventions to Reduce Stress and Burnout among Teachers: A Scoping Review

**DOI:** 10.3390/ijerph20095625

**Published:** 2023-04-24

**Authors:** Belinda Agyapong, Pamela Brett-MacLean, Lisa Burback, Vincent Israel Opoku Agyapong, Yifeng Wei

**Affiliations:** 1 Department of Psychiatry, University of Alberta, Edmonton, AB T6G 2B7, Canada; 2 Department of Psychiatry, Dalhousie University, Halifax, NS B3H 2E2, Canada

**Keywords:** teachers, stress, burnout, interventions, mindfulness, cognitive behavioral therapy

## Abstract

**Background:** Teaching is recognized as a highly challenging profession. Experience of chronic stress is a risk factor for poor mental and physical well-being, and burnout. There is limited knowledge regarding optimal interventions to address stress and burnout among teachers. **Objective:** To undertake a scoping review of the literature in the last five years to determine various psychological interventions to address stress and burnout among teachers. **Method:** The PRISMA-ScR (Preferred Reporting Items for Systematic Reviews and Meta-Analyses extension for Scoping Reviews was followed. Relevant search terms were used to determine different interventions adopted to reduce teachers’ stress and burnout. Articles published between 2018 and 2022 were identified using five bibliographic databases. Relevant articles were extracted, reviewed, collated, and thematically analyzed, and findings s were summarized. **Results:** Forty studies conducted in Asia, North America, Oceania, Europe, and Africa, met the inclusion criteria. Sixteen kinds of burnout and stress-reduction interventions were identified. The most popularly studied intervention were Mindfulness-Based Interventions alone or in combination with yoga or Cognitive Behavioural Therapy (CBT), followed by Rational Emotive Behavioral Therapy (REBT). Mindfulness-Based Interventions led to decreased overall Teacher Stress Inventory (TSI) and emotional exhaustion subscale scores. REBT, primarily used with special education teachers, especially in Africa, has also shown positive results. Other interventions reporting positive outcomes include Inquiry-Based Stress Reduction (IBSR), the Stress Management and Resiliency Training Program (SMART), Cyclic Meditation, Group Sandplay, Progressive Muscle Relaxation, Autogenic Training, Sport-Based Physical Activity, Emotional Intelligence Ability Models and Christian Prayer and Prayer-Reflection. **Conclusions:** Stress and burnout can have a negative impact on teachers and, very often, on the students they teach. Implementing suitable school-based interventions is necessary to improve teachers’ stress-coping ability, reduce the likelihood of burnout and improve general well-being. Policymakers, governments, school boards and administrators should prioritize the implementation of school-based awareness and intervention programs.

## 1. Introduction

Stress has been defined as a state of mental or emotional strain due to adverse or challenging circumstances [1]. Teaching is recognized as a high-pressure, stressful profession across the world. The term “teacher stress” was first defined by Kyriacou and Sutcliffe (1978) and refers to the experience of “negative affect (such as anger or depression) by a teacher usually accompanied by potentially pathogenic physical or biological and biochemical changes … resulting from parts of the teacher’s job ‘mediated by the perception that’ demands made upon the teacher constitute a threat to his self-esteem or well-being and by coping mechanisms activated to reduce the perceived threat” [2].

A recent scoping review reported that the prevalence of chronic stress among teachers ranged from 8.3% to 87.1%, while moderate to severe burnout among teachers ranged from 25.1% to 74.0% [3]. Teachers’ stress and burnout are reported to be influenced by several factors, including school (organizational) and work-related factors like the years of teaching, class size, job satisfaction, and the subject taught, and socio-demographic factors such as sex, age, and marital status [4,5]. In recent years, stressors experienced by teachers have only been exacerbated by the COVID-19 pandemic [6,7]. High levels of occupational stress experienced by teachers have been associated with increased absenteeism, staff turnover and a wide range of adverse health outcomes [8,9], such as fatigue, sleep disturbance, hormonal changes [10], and elevated rates of burnout, anxiety and depression. These can negatively affect teachers’ personal lives, work-related performance, and productivity, thus inadvertently impacting students [8,11]. As cumulative stress may lead to burnout and, subsequently, anxiety and depression, interventions focused on helping teachers to manage stress may enhance the health and well-being of teachers and reduce healthcare costs [3,8,12]. Considering the high levels of chronic stress and burnout experienced by teachers and its associated negative impacts, it is important to examine and assess interventions that have been introduced to address these psychological issues among teachers.

A previous systematic review [13] by von der Embse et al. (2019), which included 24 articles published from 1998–2017, suggested that the most effective interventions were in the mindfulness, behavioral, and cognitive-behavioral domains, while interventions solely delivering solely informational content were among the least effective. Another review [14] conducted by Sanetti et al. (2021) reported on 18 articles published from 1987–2016 and indicated that the most commonly evaluated stress-reduction intervention incorporated meditation or mindfulness-based practices. A systematic review that focused on the impact of the COVID-19 pandemic found that many workers who shifted to working from home via telework experienced physical and mental stress [15]. One cross-sectional study [16] indicated that half of the respondents self-reported a decrease in their well-being at work and increased stress levels. However, in another study [17], it was reported that teachers had less difficulty with the teaching-learning model changes and how they dealt with confinement during the pandemic. The shift away from having to physically deal with students’ discipline and attendance issues to other needs may have been a factor. Similar conclusions were noted in a study [16] investigating the impact of COVID-19 on childcare centers. This study reported that educators working remotely were more likely to report a lower level of stress than when working with children at daycare (36.1% vs. 19.7%). This scoping review gives a current overview (2018 to 2022) of the interventions published in the last five years that were adopted to combat stress and burnout among teachers, which includes relevant literature during the COVID-19 pandemic. This scoping review aims to determine the different modes of psychological interventions adopted specifically for teachers and to summarize their reported effectiveness for reducing stress and burnout. The specific research question was: What interventions have been used to reduce stress and burnout among teachers, and what was their reported effectiveness? The review will identify the gaps in knowledge related to interventions to address stress and burnout among teachers and identify opportunities for future research. This manuscript includes the following sections: methods, how the scoping review was conducted, results, discussion, implications for policy and practice and future research directions, limitations, and conclusion.

## 2. Methods

### 2.1. Study Design

This review was conducted in adherence to the Preferred Reporting Items for Systematic Reviews and Meta-Analyses Extension for Scoping Reviews (PRISMA-ScR) statement [18]. A comprehensive search strategy that allows replicability, reliability, and transparency was adopted. The review followed Arksey and O’Malley’s five-stage approach to scoping reviews [19], including developing the research question, searching for relevant studies, selecting articles, data charting and data extraction and collating, summarizing and reporting the results.

### 2.2. Identifying Relevant Studies

A systematic literature search of several electronic bibliographic databases, including the following databases: CINAHL (Cumulative Index of Nursing and Allied Health Literature) Plus with Full Text (EBSCOhost interface), PubMed (Public/Publisher MEDLINE (NLM journal articles database), APA PsycINFO (Ovid interface), MEDLINE (Medical Literature Analysis and Retrieval System Online), and Scopus Elsevier was conducted using relevant terms to identify and select articles. The search consisted of keywords representing the concepts of interventions to mitigate stress and burnout among teachers. The specific MeSH terms, keywords and descriptors included (teacher* or “school teachers” or educators or “school staff” or tutor* or schoolteacher or teach*) AND (stress or “mental exhaustion” or “psychological stress” or “emotional exhaustion” or burnout or “burn out” OR burnout) AND (“intervention*” OR therapy OR management OR treatment OR intervention OR interventions or strategies or techniques or management OR “psychological treatment” OR “teachers stress management technique” or “teacher burnout management techniques” OR “teacher stress intervention” OR “teachers burnout interventions”). Publication year restrictions were applied (2018 to 2022) to ensure only current interventions are captured. This scoping review builds on previous reviews of the literature, for instance, by Von der Embase et al., 2019 and Hagermoser et al., 2021 [13,14], extending understanding of this growing area of literature.

### 2.3. Articles Selection

Two researchers independently reviewed the citations during the title, abstract screening, and full-text review phase based on specific eligibility criteria. All discrepancies were resolved through discussion and consensus. Articles were eligible for inclusion only if they discussed interventions to reduce burnout or stress among classroom, special education, primary, elementary, middle, and secondary school teachers and educators. Meta-analyses, systematic reviews, case reports, opinion pieces, commentaries, editorials, or grey literature such as non-peer-reviewed graduate student theses, non-research articles or conference reports were excluded. Articles were limited to original, peer-reviewed articles written in English. Articles were excluded from the review if the study focused only on correlates of or prevalence of stress and burnout. Articles were also excluded if study participants included preschool teachers or tertiary educators working in vocational, adult or community continuing education settings, university teachers, students, or a combination of teachers and students.

**Figure 1:**
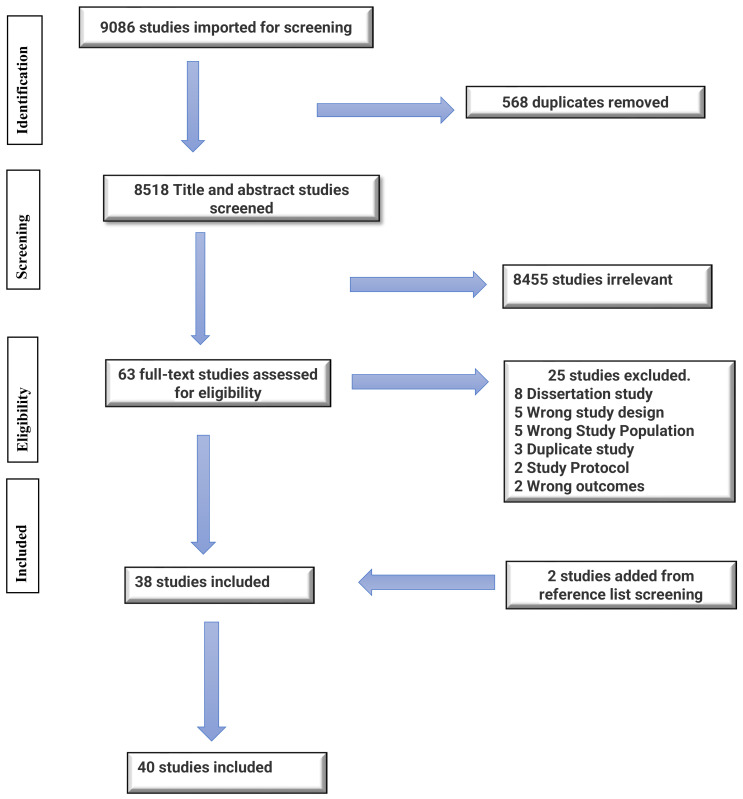
PRISMA flow chart.

We identified 63 articles for full-text review but excluded 25 articles that did not meet the inclusion criteria on closer examination. A review of the reference list from other studies was also explored and yielded two additional eligible articles, which were included in this scoping review. The PRISMA flow diagram presented in Figure 1 gives comprehensive details of this information.

### 2.4. Data Charting and Extraction

The research team extracted the following information from each selected article according to the following domains: author(s) name, year of publication, country of study, study design, intervention (focus and content), study procedures, participants; sample size (N), participant age range, assessment measures used, and key findings.

### 2.5. Collating, Summarizing, and Reporting the Results

This scoping review summarizes recent evidence regarding interventions that have been used to reduce stress and burnout among teachers. All the relevant data were organized into tables and validated by at least two team members. The characteristics and results reported in each included article were summarized.

## 3. Results

**Figure 2:**
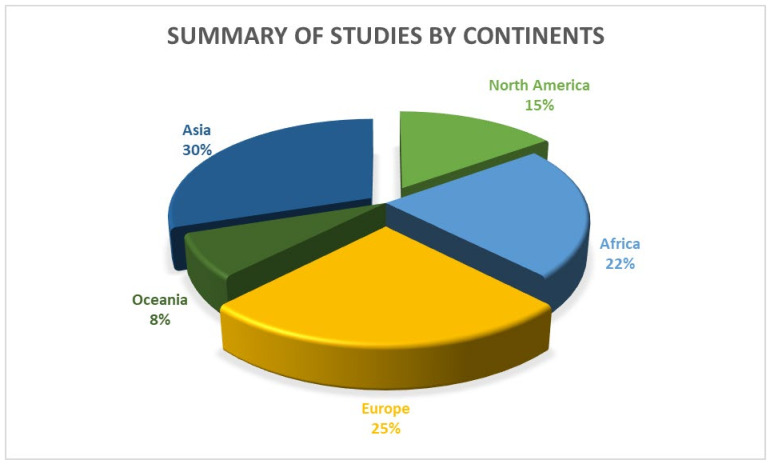
Summary of studies by continent.

The database search was completed on 10 December 2022 and included results from 2018 to 2022. A total of 9086 records were identified through the database searches. Five hundred sixty-eight (568) duplicate records were identified and removed automatically when imported into the systematic review management software, Covidence [20]. The characteristics of the 40 articles in this scoping review are presented in Table 1. Most of the studies (n = 20, 50.0%) were non-randomized controlled trials (Non RCTs), Sixteen studies (n = 16, 40.0%) were simple RCT or RCT waitlist or group-randomized waitlist control (WLC) trials, and the remaining four were (n = 4, 10.0%) cluster RCT’s. The 40 articles included a total of 4344 participants. The sample size for the individual articles ranged from 24 to 672 participants, with an age range from 21 to 70 years. Most studies (77.0%) were published between 2020 and 2022, and most of the studies were conducted in Asia (30.0%), followed by Europe (25.0%), then Africa (22.0%) and North America (15.0%), and Oceania (8.0%) as shown in Figure 2.

**Figure 3:**
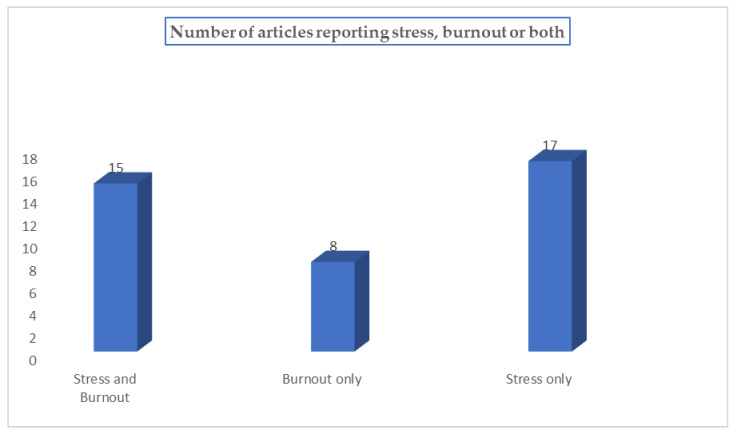
The number of articles reporting on stress and burnout.

From Figure 3, 15 out of 40 articles (37.5%) focused on reducing both burnout and stress among teachers, 16 articles (40.0%) focused on stress, and nine articles (22.5%) focused on burnout.

**Figure 4:**
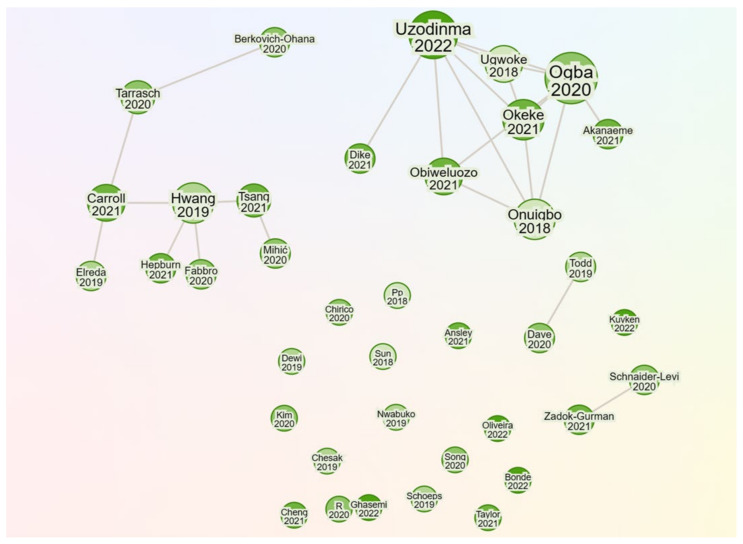
Visual network of included studies (authors and year of publication).

Figure 4 shows a visual network of the individual studies. The visual network was created by inputting all the studies included in this scoping review into the ResearchRabbit online application [21].

Figure 4 suggests that Uzodinma 2022 and Okeke 2021 are each connected by citation to five of the included articles, whilst Hwang 2019, Ogba 2020, and Onuigbo 2018 are each connected by citation to four of the included studies. In addition, Ugwoke 2018, Obiweluozo 2021, and Carroll 2021 are each connected by citation to three of the included articles, and Tsang 2021 and Tarrasch are connected by citation to two of the included articles. Finally, Mihic 2020, Dave 2020, Todd 2019, Zadok-Gurman 2021, and Scnaider-Levi 2020 are each connected by citation to only one of the included studies in this scoping review. The remaining other studies, including Nwabuko 2020, Chesak 2019, Chirico 2020 and Song 2020, have no connections by citation with any other study included in the scoping review. Most of the visually highly connected studies were conducted in Africa with a sample of teachers who teach special needs students.

### 3.1. Scales Used to Measure Stress and Burnout

The Perceived Stress Scale (PSS-10) was used to assess stress in 16 articles; the Depression, Anxiety and Stress Scales (DASS-21) in six articles; the Teacher Stress Inventory (TSI) in six articles; the Single Item Stress Questionnaire (SISQ) in four articles; the Perceived Occupational Stress Scale in 2 articles; the Teacher stress questionnaire (TSQ), in one article; and the Coping Style Questionnaire and the Teacher Stress Question were used in one article each.

Burnout was assessed using the Maslach Burnout Inventory—(both the “Educators Survey” (MBI-ES) and the “General Survey” (MBI-GS) versions) in 18 articles, the Shirom-Melamed Burnout Measure (SMBM) in two articles, and the Copenhagen Burnout Inventory (CBI), Spanish Burnout Inventory and the Teacher Burnout inventory (TBI), in one article each.

### 3.2. Interventions to Reduce Stress and Burnout among Teachers

This scoping review identified several interventions (summarized in Table 1, Table 2 and Table 3) that have been adopted to alleviate teachers’ stress and burnout.

**Table 1:** Mindfulness-based interventions, CBT, and Yoga, either alone or in combination.

Authors/Year	Country (Continent)	Study Design	Intervention: Focus and Content	Study Procedures	Participants, Sample Size	Age (Range, Mean, SD)	Scales Used to Measure Primary Outcome	Key Findings
Akanaeme et al., 2021 [22]	Nigeria (Africa)	Randomized control trial (RCT), with “waitlist” control (WLC)	Focus: Stress Content: Yoga and CBT (Y-CBT) program. (12 weekly 2-h sessions including meditation practices and breathing exercises).	Participants were randomly assigned to a Y-CBT group (n = 29) or WLC group (n = 29). Pre-, post- and follow-up outcome assessment.	Special education teachers, n = 58	Y-CBT: mean age = 31.8 years. WLC: mean age = 32.1	Single Item Stress Question (SISQ); Teacher Stress Inventory (TSI)	Improvement in TSI score of Y-CBT and WLC groups at post-test, (*p* = 0.00), and follow-up. (*p* = 0.00). Improved SISQ ratings in the Y-CBT participants compared to the WLC group.
Ansley et al., 2021 [23]	USA (North America)	RCT	Focus: Burnout Content: 8 self-paced, 30 min, asynchronous, online modules on occupational wellness, self-care, mindfulness, and cognitive restructuring.	Participants were randomly assigned to the online IG or CG. Self-report scales collect pre-, and post-intervention information.	Teachers (e.g., Special education, general education teachers, paraeducators etc., n = 51)	Age ≤ 24 years (31%); 25–34 years (49%); 35+ years (40%)	Maslach Burnout Inventory—Educators Survey (MBI-ES)	IG experienced improvement in coping ability, efficacy, and P.A, although PA increases were not significant. IG also experienced significant decreases in E.E. (*p* = 0.05) and D.P (*p* = 0.07).
Bonde et al., 2022 [24]	Denmark (Europe)	Cluster RCT waitlist	Focus: Stress Content: Mindfulness-Based Stress Reduction (MBSR) is a curriculum-based intervention consisting of 8 weekly 2.5 h sessions, 7 h silent retreat, and daily mindfulness practice (1 h, 6 days per week).	Teachers were cluster randomized to the first offering of MBSR program (in 2019) or WLC group (which received the intervention in 2020).	Teachers n = 191	Mean age 45.2 years	Perceived Stress Scale (PSS-10)	At 3 months PSS scores were lower for the IG compared to WLC participants. Similar results were obtained at 6 months for the CG.
Carroll et al., 2022 [25]	Australia (Oceania)	Non RCT: Matched sample longitudinal design	Focus: Stress, Burnout Content: Mindfulness-Based Stress Reduction Intervention (MBSR). 8 weekly 2.5-h group session, daily home practice, and a full day retreat in week 5 or 6.	Participants were assigned to either the (MBSR group; n = 42) or Health Enhancement Program (HEP group; n = 41), Participants were assessed across three time points:	75 Teachers	Age range: 22 to 69 years Mean age = 45.28 (SD = 11.5)	Perceived Stress Scale (PSS-10). Copenhagen Burnout Inventory (CBI). Depression Anxiety Stress Scales (DASS–21)	Both MBSR and HEP resulted in reduced perceived stress from pre-to post-intervention and follow-up. PSS scores (*p* ≤ 0.001). DASS stress, *p* ≤ 0.001, and work burnout, (*p* < 0.001) with large effect sizes.
Cheng et al., 2022 [26]	China (Asia)	Non RCT: Quasi-experimental (mixed methods) design	Focus: Stress; Burnout Content: 4 sessions, weekly mindfulness training (MT) intervention included MBSR and Mindfulness-Based Cognitive Therapy (MBCT exercises).	Participants were assigned to the MT IG s (n = 35, comparison group n = 35, qualitative group n = 24)	kindergarten teachers n= 70 qualitative n = 24	Age range 21 to 50 years. Mean age = 30.96 years (SD = 6.7).	Depression, Anxiety and Stress Scales (DASS-21). Maslach Burnout Inventory (MBI- GS).	Reduction in MT and comparison group Stress; DASS total score *p* = 0.03, and burnout *p* = 0.02 Depersonalization *p* = 0.02 No significant differences were found for EE *p* = 0.2, Lack of PA *p* = 0.08
Dave et al., 2020 [27]	USA (North America)	Non RCT: Longitudinal cohort design	Focus: Burnout Content: Inner Journey Mindfulness-Based Stress Reduction (IJ-MBSR): an original MBSR, eight 2.5–3-h classes sessions, a day silent retreat, 45 min of daily structured practice. the IJ-MBSR format emphasized loving kindness and included elements of MBCT.	The first two cohorts (n = 78) completed the IJ-MBSR. n = 158.	236 (Private and public K-12 Teachers)	Age range 22 to 68 years Mean age = 48.8 years (SD = 11.4)	Maslach Burnout Inventory—Educator Survey (MSB-ES)	Statistically significant differences were found in two components of burnout; EE decreased, (*p* = 0.001), and P.A increased (*p* = 0.001).
Dike et al., 2021 [28]	Nigeria (Africa)	Group-RCT	Focus: Stress, burnout Content: Yoga and Cognitive Behavioral Therapy (Y-CBT). 12 weekly 2-h, sessions.	Participants were randomly assigned to Y-CBT (n = 29) or WLC (n = 29) groups. Baseline, post-test, and follow-up assessment.	Special education teachers, n = 58	NR	Single Item Stress Questionnaire (SISQ); Maslach Burnout Inventory—Educators Survey (MBI-ES)	Y-CBT group reported significant reduction in overall MBI-ES scores across Time 1 and 2; (*p* = 0.00); but not significant across Time 2–3; (*p* = 0.21). No significant changes in MBI-ES scores in the WLC group across Time 1–2, *p* = 0.12, and Time 2–3 *p* = 0.1
Fabbro et al., 2020 [29]	Italy (Europe)	Non RCT: Prospective, controlled longitudinal study.	Focus: Stress, Burnout Content: Mindfulness-Oriented Meditation (MOM): 8 weekly sessions (no day-long mindfulness retreat).	Teachers were assigned to a MOM group (n = 19) or a WLC group (n = 20).	Female Teachers n = 39	MOM: age range 28–63 Mean age 51.5 (SD = 9.2) CG: age range 34–60 Mean age = 50.2 (SD = 8.28)	Teacher Stress Inventory (TSI); Maslach Burnout Inventory—Educators Survey (MSB-ES)	Teachers in the MOM group had lower stress and burnout levels compared to the WLC group. *p* = 0.04. Post-hoc test showed decrease of TSI scores in the MOM group (*p* = 0.01) EE subscale, (*p* = 0.02), and (*p* = 0.06) respectively.
Ghasemi et al., 2022 [30]	Iran (Asian)	RCT	Focus: Burnout Content: Group-based cognitive–behavioral therapy (CBT) program (8 weeks-session)	Teachers were randomly assigned to either a group- CBT program or a WLC group. Multiple assessments at pre-treatment, post-treatment, and 6-month follow-up.	Teachers n = 62	Mean age = 31.5 years (SD = 4.6)	Maslach Burnout Inventory—Educators Survey (MBI-ES)	Improved outcomes for treatment group compared to the WLC on the three subscales of the MBI-ES (E.E, D.P, and reduced P.A) scores (teachers at post-treatment, with improvement maintained at 6-month follow-up
Hepburn et al., 2021 [31]	Australia (Oceania)	Non RCT: mixed methods design	Focus: Burnout, Stress Content: 6 weekly sessions of an intervention focused on strategies and techniques for enhancing awareness and regulation of the stress response through cognitive and physiological mechanisms.	Self-selected participants registered for the study. Both qualitative and quantitative. Multiple assessments: pre-, post-, and 3-month follow-up.	Teachers n = 24	Age range 23 to 58 years. Mean age = 36.9 (SD = 11.7).	Perceived Stress Scale (PSS-10); Maslach Burnout Inventory—Educators Survey (MBI-ES).	IG: a significant decrease in perceived stress scores. There was also a decrease in MSI-ES, E.E, PA and D.P subscales scores.
Hwang et al., 2019 [32]	Australia (Oceania)	Cluster RCT design	Focus: Stress Content: Mindfulness-based intervention (MBI) including yoga. 8 weekly 1.5 hrs. organized at 10 schools.	Teachers were randomized to an IG or a CG MBI was implemented at 10 schools. The same intervention I was provided at 10 schools in the CG during the third school term.	Teachers n = 185	IG: mean age = 42.3 years CG: mean age = 43.7 years.	Perceived Stress Scale (PSS-10)	IG had lower levels of perceived stress (*p* < 0.01).
Kuyken et al., 2022 [33]	England (UK) (Europe)	Cluster RCT	Focus: Stress, Burnout Content: Universal School-Based Mindfulness Training (SBMT). 8-sessions Mindfulness-Based Cognitive Therapy for life (MBCT-L) program. Each session was 2 h a week and 40 min per day of mindfulness practice.	Teachers from 85 schools were randomized to either teaching as usual (TAU) or include universal SBMT. 8-week personal	Teachers n = 672	SBMT: mean age 40.2 years (SD 8.9) TAU: mean age 39.1 years (SD 9.2)	Maslach Burnout Inventory—Educator Survey (MBI-ES); Perceived Stress Scale (PSS-10)	EE (−0.22; 95% CI −0.38 to −0.05); PA (−0.21; −0.41, −0.02); Effects on burnout were not significant at 1-year follow-up. SBMT supports short-term changes in teacher burnout
Maratos et al., 2019 [34]	England (UK) (Europe)	Non RCT: Mixed methods	Focus: Stress, Burnout Content: 6 sessions, each lasting about 2.5 h compassionate mind training (CMT) program	The psychoeducational aspect was followed by the introduction of two short exercises. Quantitative and qualitative design outcome measures collected 2 months prior to first session—T1, one week prior to—T2, and one-month post-intervention—T3; qualitative analysis of implementation (post-intervention focus group).	Teachers and support staff, n = 70	T1- Mean age = 40.5 years T2- Mean age = 38.1 years T3- Mean age = 36.1 years	Depression, Anxiety and Stress Scales (DASS-21). Maslach Burnout Inventory (MBI-GS)	EE significantly decreased from T1 to T2 (*p* = 0.03). P.A significantly increased from T1 to T2, (*p* = 0.03). No significant differences with respect to E.E or P.A were obtained at T3 compared to T2 (*p* > 0.1).
Matos et al., 2022 [35]	Portugal (Europe)	RCT (waitlist)	Focus: Stress, Burnout Content: 8 weekly sessions, 2.5 h each Compassionate Mind Training program for Teachers (CMT-T)	Teachers were randomized to a CMT-T group or a WLC group.	Public school teachers n = 155	Age: range 25 to 63 years Mean age = 51.4 (SD = 7.2).	Depression, Anxiety and Stress Scales (DASS-21). Shirom-Melamed Burnout Measure (SMBM).	CMT-T group: significant decrease in burnout from T1 to T2. WLC participants who received CMT-T showed decreases in burnout and stress. Burnout (*p* = 0.036) All differences reflected large effect sizes.
Mihic et al., 2020 [36]	Croatia (Europe)	Non RCT, Prospective controlled longitudinal study	Focus: Burnout Content: Mindfulness-Based Social-Emotional Learning Program CARE 30 h over five in-person training days (6 h each). 2-day weekend session (12 h) followed by a 2-day session a week later. Then a 1-day booster session	The CARE for Teachers training. n = 25 received CARE f and n = 29 was in the control condition.	Teachers and school personnel from public schools n = 54	Age range23 to 63 Mean age = 42.55 (SD = 10.63).	Maslach Burnout Inventory—MBI	There were no effects of the intervention on burnout measures at follow-up.
Molloy Elreda et al., 2019 [37]	USA (North America)	Cluster RCT	Focus: Stress Content: Mindfulness-Based Emotion Skills Program. Interpersonal mindfulness. Each observation was composed of three 22-min cycles, including 15 min of observing CLASS indicators and 7 min of coding.	Trained, independent researchers used the K-3 CLASS to observe and assess participants on two separate days within the same week for approximately an hour each day while the target teacher was leading the class.	Teachers n = 224	Age range 22 to 73 years.	Perceived Stress Scale (PSS-10)	Significant relation between teachers’ perceived stress and teachers’ emotional supportiveness in the classroom (*p* = 0.04). Interpersonal mindfulness served as a protective factor for teachers at high levels of stress.
Song et al., 2020 [38]	China (Asia)	Non RCT: Group comparison study	Focus: Stress Content: Mindfulness Training (MT) 4-day intensive program. The training course delivered by the therapist lasted 8 h from 9 a.m. to 5 p.m. with 2 h midday rest on each day.	Teachers voluntarily signed up to participate in different monthly courses depending on their work schedules, mindfulness group, April course, or the waitlist group July course.	Primary, middle, and high school public teachers n = 161	Age range 24 to 55 years Mean age = 38.5 years (SD = 6.8)	Perceived Stress Scale (PSS-10), Chinese version	MT program decreased teachers’ stress and improved emotional health.
Tarrasch et al., 2020 [39]	Israel (Asia)	Random/passive control trial	Focus: Stress, burnout Content: “Call to Care—Israel for Teachers” (C2CIT) program utilizes mindfulness, compassion, and social-emotional skill training, and self-care. 20 weekly meetings. Each session in the 3 modes lasted 90 min.	Teachers were allocated into either the C2CIT program, or passive control. Sessions included psychoeducational materials.	Middle school teachers n = 44	Age range 28 to 52 Mean age = 34.9 (SD = 7.9)	Perceived Stress Scale (PSS-10), Maslach Burnout Inventory (MBI)	Distress, perceived stress, and self-compassion scores improved for teachers in the C2CIT group. Significant change in perceived stress, *p* < 0.001 in C2CIT group. C2CIT vs Passive Control: E.E: *p* = 0.85, *p* = 0.068 D.P: *p* = 0.47, *p* = 0.53 Reduced P.A: *p* = 0.61, *p* = 0.29
Taylor et al., 2021 [12]	USA (North America)	Randomized waitlist-control design.	Focus: Stress, Burnout Content: Brief Mindfulness-Based Interventions (bMBI). Four total sessions; six total contact hours. Duration, 16 weeks and included one 90-min session per month.	Teachers were randomly assigned to either the IG (n = 12) or WLC (n = 12) group and received the intervention respectively (January to June) and the Fall (August to November) semester of 2018.	High school teachers n = 24	Age range 25 to 70 Mean age = 42.8	Maslach Burnout Inventory—Educators Survey (MBI-ES); Teacher Stress Inventory (TSI)	Significant reductions in teacher stress for the IG from pre-to post intervention *p* = 0.001 and work-Related Stressors, *p* = 0.01 Significant reductions in symptoms of burnout from pre-to post-intervention (*p* = 0.01) Significant reductions from pre-to post-intervention on the EE; *p* = 0.003 but not DP or PA.
Todd et al., 2019 [40]	Wales (UK) (Europe)	Non RCT: Mixed methods “natural experiment evaluation”.	Focus: Stress Content: Mindfulness-Based Stress Reduction (MBSR). 8-week, 2 h per week MBSR course focused on experiential learning, and 8-week, 1.5 h per week informational-focused mindfulness course (“Foundations”)	Comparison of a MBSR and informational-focused mindfulness course (“Foundations” courses)	Primary and secondary school teachers; n = 69	MBSR- Age range 28 to 61 years Mean age = 42.5 years Foundation Course Age range 24 to 58 years Mean age = 40.	Perceived Stress Scale (PSS-10)	Both courses were associated with significant reductions in stress.
Tsang et al., 2021 [41]	China (Asia)	RCT (waitlist)	Focus: Stress Content: Mindfulness-Based Interventions—“Foundations course”. 8 weekly 1.5 h sessions based on MBSR and MBCT, with audio guides to support 20 min daily practice.	Teachers were randomly assigned to mindfulness training (eight-week Foundations) or WLC condition. Multiple assessments.	Primary and secondary school teachers n = 186	Age range 22 to 59 years Mean age = 39.6. (SD = 9.4)	Perceived Stress Scale (PSS-10)	IG reported significantly higher levels of life satisfaction, positive affect and general health. IG had significantly lower levels of stress than the WLC at post-test and 2-month follow-up. The effect sizes were medium to large.
Berkovich-Ohana et al., 2020 [42]	Israel (Asia)	Non-RCT Convenience sampling	Focus: Stress Content: MBI course “Applied Mindful Pedagogy for Educators” 10-session (30 h, 3-month course) Mindful Self in School Relationships (MSSR) model. cognitive intervention	MBI group completed a 30 h, 6-month training course called Teaching for Understanding on Constructivist Pedagogy. Multiple assessments.	Elementary school teachers, n = 39	NR	Perceived Stress Scale (PSS 10)	Significant decrease in stress in the MBI group between T1 and T3 (*p* < 0.01). There was also a significant difference between the groups at T3; (*p* < 0.05)
Dahal and Pradhan 2018 [43]	Nepal (Asia)	Non-RCT	Focus: Stress Content: Cyclic Meditation (CM) Deep Relaxation Intervention was held for 1 month and combines physical postures and movement with relaxation procedures—20 daily 30 min sessions over one month.	Pre- and post-intervention comparison.	High school teachers n = 62	Age range 25 to 55 years. Mean age = 37.4 (SD = 8.6).	Perceived Stress Scale (PSS 10)	Significant reduction in stress (PSS stress scores (*p* < 0.001) after the intervention.)

TSI—Teacher stress inventory, DP—Depersonalization, PA—Personal accomplishment, EE—Emotional exhaustion, Y-CBT—Yoga-Based Cognitive Behavioral Therapy, CBT—Cognitive Behavioral Therapy, MBI—Mindfulness-Based Intervention, MBSR—Mindfulness-Based Stress Reduction, WLC—Waitlist control, RCT—Randomized control trial, NR—Not reported, IG—Intervention group, CG—Control group, CASS—Classroom Assessment Scoring System, SD—Standard Deviation, h (s)—Hour (s).

This review found 40 articles with sixteen types of interventions for addressing burnout and stress in teachers: (a) 18 studies on mindfulness-based interventions [12,23,24,25,27,29,33,34,35,36,37,38,39,40,41] including in combination with yoga [32] or in combination with Cognitive Behavioural Therapy (CBT) [26,42], (b) seven studies reported on CBT only [30,31] or CBT in combination with yoga [22,28], or other derivatives, e.g., Inquiry-Based Stress Reduction (IBSR) [57,60] and Cyclic meditation [43] (c) seven studies used Rational Emotive Behavior Therapy [44,45,46,47,48,49,50], (d) one study on Sports-Based Physical Activity Program [55] (e) Christian prayer and prayer-reflection [52] (f) Group Sandplay [58], (g) stress reduction training (e.g., Stress Management and Resiliency Training Program (SMART) [51], Autogenic Training [59], Progressive Muscle Relaxation with music and aromatherapy [53]), and (h) interventions focusing on building social and emotional competence (e.g., A+ intervention [56], Ability Model of Emotional intelligence [54].

### 3.3. Mindfulness and Mindfulness-Based with CBT or Yoga Interventions

Mindfulness is the most popularly utilized intervention studied to reduce stress and burnout among teachers, and most studies reported high efficacy [25,27,29,38], with a significant decrease in Teacher Stress Inventory (TSI) scores and emotional exhaustion subscale. However, one study reported no change in the depersonalization subscale of burnout [27]. Most mindfulness-based interventions are time intensive; Mindfulness-Based Stress Reduction (MBSR), for example, includes eight weekly 2.5 h sessions and a 7 h or full day retreat [24,32], and Compassionate Mind Training (CMT) program extends for 12 weeks [34]. Yoga-based practices for teachers complement some mindfulness-based interventions, with reported lower levels of perceived stress [32].

CBT-based interventions resulted in statistically significant improvements in perceived stress [31] and MBI-ES subscales for emotional exhaustion and depersonalization [30,31] and personal accomplishment [30]. Yoga in combination with CBT (Y-CBT) involved sessions of physical/posture exercises and meditation practices associated with reductions in perceived stress and burnout [22,28].

### 3.4. Rational Emotive Occupational Health Coaching (REOHC)

Rational Emotive Occupational Health Coaching (REOHC), though similar to CBT, focuses on rational thinking and positive regard for the self. It involves a 12-week of 2 h weekly stress management program and has been employed mostly among teachers of children with special needs, including children with autism [46,48,49]. REOHC significantly decreased job burnout and emotional exhaustion among special needs teachers [46,48,49].

### 3.5. Inquiry-Based Stress Reduction (IBSR) Intervention

This intervention is a cognitive-reframing program and includes 3.5 h weekly group meetings and weekly individual sessions with a facilitator (1 h/session) for 12 weeks [57]. The outcome of one study indicated that teachers in the intervention group showed greater improvements in emotional exhaustion (*p* = 0.01) and personal accomplishment (*p* = 0.04) compared to controls [57]. This contradicts the outcome reported by Zadok-Gurman et al., which suggested no difference in personal accomplishment scales between the intervention and control groups [60].

### 3.6. Other Interventions

Other interventions to decrease teachers’ stress and burnout include the A+ intervention [56], sports-based physical activity program [55], the ability model of the emotional intelligence training program [54], progressive muscle relaxation with music and aromatherapy [53], Christian prayer and prayer reflection [52], Stress Management and Resiliency Training Program; SMART [51], group sandplay [58], and Autogenic training [59].

The A+ intervention had reported positive impacts on emotional well-being, occupational stress and emotional exhaustion symptoms in one study [56]. The sports-based physical activity program [55] comprises seven weeks of training in catchall, a team sport and a tournament as the culminating event. Qualitative results suggested this program helped decrease teachers’ stress levels. However, quantitative results indicated no significant pre–post main effect on the Perceived Stress Scale (PSS-10) scores. The ability model of emotional intelligence [54] training program consisted of five two hours sessions for three months. The effect size was moderate to high, but the differences at Time 2 were only partially maintained at Time 3, and multivariate tests indicated only marginal significance for burnout (*p* = 0.06). In addition, no significant differences were found for emotional symptoms (*p* = 0.31). However, the intervention group experienced reduced levels of work-related stress and emotional symptoms compared to the control group. Progressive muscle relaxation with music and aromatherapy [53] included four 20 min therapy sessions over four days. Participants in the intervention group reported a significant reduction in the Teacher Stress Inventory, compared to no change in the control group. The Christian prayer and prayer reflection [52] involved two 30 min training sessions a week over two months. This intervention is a combination of individual Christian prayer and a focus group of prayer reflection. Teachers who received this intervention reported significant improvement in emotional exhaustion (*p* < 0.001) and depersonalization (*p* < 0.001) levels. SMART [51] program consists of an initial 90-min introductory session, followed by 12 online, self-paced modules, which participants complete at their convenience and desired pace. Weekly emails were also sent to the participants during weeks 8 to 52 of the study. In addition, eight one-hour teleconferences were provided at regular intervals. This program was associated with significantly lower stress at each follow-up survey (2, 6, and 12 months) compared to baseline (*p* = 0.003). Group sandplay [58] engaged participants in group sandplay activities where they were given opportunities to create scenes and themes. These are then analyzed, and problems regarding work-related stress scenarios are solved while receiving social support from the other group members. This study reported significant improvement in the experimental sandplay group, with a shift from passive coping pre-test to active coping post-test, suggesting that group sandplay improved overall stress-coping abilities. The final intervention showing a reduction in work-related stressors (*p* = 0.05) was Autogenic training [59]. This relaxation training technique involves six progressive steps practiced for 15–20 min while concentrating on breathing and relaxing the muscles.

## 4. Discussion

Stress and burnout among teachers can negatively impact their capacity to perform job functions, productivity and their ability to build positive relationships related to their role [8,61]. This is an important global problem, given the connection between stress and burnout and the subsequent development of anxiety and depression among teachers, as highlighted in a recent scoping review [8]. Thus, interventions designed to reduce stress response, burnout, associated negative beliefs and other aspects of functioning may be essential to reducing teachers’ anxiety and depression. Identifying effective interventions for addressing stress and burnout among teachers is a vital initial step in dealing with this global problem. This scoping review identified 40 studies examining interventions to reduce or mitigate teachers’ stress and burnout. Moreover, effective coping, which may be improved during such programs, is an instrumental skill set that ultimately benefits the teachers’ role [62]. Although most interventions intended to reduce teachers’ stress and burnout have had limited success [9], this scoping review found that some interventions can potentially address stress and burnout among teachers.

Mindfulness is an attribute of consciousness and can play a critical role in promoting the psychological well-being of teachers [63,64]. Mindfulness has been used across several fields of health to prevent or reduce stress and burnout symptoms [65]. In addition, mindfulness in teachers has been reported to show a negative association with negative emotional states such as burnout (emotional exhaustion) [63]. In clinical practice, mindfulness-based interventions have been found efficacious for depression and anxiety and placed in the same category as CBT [66,67]. Mindfulness-based interventions have also shown small-to-medium positive effects on therapeutic processes and therapeutic outcomes [9] and had a medium treatment effect on teacher outcomes and an inverse relation with teachers’ psychological distress [9,63]. In other professionals, such as nurses, mindfulness meditation has also decreased stress and burnout [68,69]. Most mindfulness-based interventions require time to practice and learn [24,32,34]. Teachers already experience high levels of stress associated with intense job demands even when working conditions are optimal [70], which may make these interventions challenging to access, especially during the school term. Researchers have proposed a 4-day intensive mindfulness training program [38], which may have higher acceptance and feasibility than the standard 8-week training program. A program with a shorter duration may improve engagement, and hence teachers may benefit from participating. This may be a promising way to decrease teachers’ stress and improve their emotional health.

There were very few online or computerized interventions. Online interventions may be more accessible and flexible for teachers who already have busy schedules, improving feasibility. In addition, this may improve access and address the needs of those in remote areas [71]. For instance, the SMART intervention [51] was an online-based program that eliminated the face-to-face nature of other interventions and positively impacted teacher stress and burnout [32]. SMART intervention [72] has also been employed among nurses resulting in statistically significant decreases in stress and burnout and increases in resilience. However, SMART consists of 12 modules, requiring significant time to complete [51,72].

CBT is based on the theory that dysfunctional thoughts and beliefs are the main drivers of distress and that thoughts, behaviors, and emotions all impact each other. CBT has been adopted in several healthcare sectors [73], with proven benefits for various psychiatric disorders, including depression, anxiety, and personality disorders [74]. For instance, a group-based CBT program [30] reported significantly greater improvements, compared to the control condition, on the total burnout subscales scores (emotional exhaustion, depersonalization, and reduced personal accomplishment) at post-treatment, with treatment effects maintained at 6-month follow-up. REBT, similar to CBT, has also demonstrated positive impacts on stress and burnout, among special educators [46,47,48] and in the health sector, in addition to reducing depression in adults with congenital heart disease [75].

In contrast, yoga is a complementary mind-body intervention with a limited focus on cognition and yet has also been found helpful in reducing psychosomatic challenges such as stress and burnout in the clinical sample [76]. Interestingly, the combination of yoga-based CBT interventions among teachers [22,28] has been reported to be effective in reducing stress and burnout, perhaps by combining benefits derived from both cognitive and body-based foci. Nonetheless, this intervention is also time-consuming, involving two hours weekly program for 12 weeks, which may create excess time demands, contributing to additional stress. Stress is presumed to occur when a person perceives an external demand as exceeding their capability [77]. Thus, teachers, when work or time demand exceeds their capability, then they may be stressed. Therefore, future research should therefore focus on teachers teaching different groups of students with different abilities, socioeconomic backgrounds and special needs to address the research gaps arising from differences in the effects of interventions to mitigate stress and burnout. Furthermore, some interventions involving physical activities [22,55] may be inaccessible to individual teachers with mobility or morbidity issues.

Christian prayer and prayer-reflection interventions [52] are novel and have rarely been used as a management strategy for stress or burnout in other professions. However, exploring the spirituality of physicians through a survey, respondents indicated barriers to time and training [78]. Christian prayer and prayer-reflection intervention [52] seemed to have a significant positive effect on depersonalization, unlike brief mindfulness-based interventions [12], which showed no significant effects for either the depersonalization subscale or the personal accomplishment subscale. However, not all teachers may be willing to access this intervention due to its faith-based nature. Depersonalization or cynicism is the interpersonal dimension of burnout and is associated with the negative, callous, or excessively detached response to other people [79]. Prayer/spiritual intervention may relieve teachers of the subscale depersonalization by helping them to focus on something larger than themselves and address some of the beliefs or narratives that might contribute to depersonalization. This speculation remains to be tested empirically.

Unlike Christian prayer and prayer-reflection [52], Blended Inquiry-Based Stress Reduction (IBSR) technique does not require any religious or spiritual preparation or intellectual ability but rather one’s desire to deepen self-awareness [60,80]. Compared to other interventions [34,55,58], IBSR does not require a trained facilitator. IBSR [60] resulted in no change in the personal accomplishment scales in the intervention group compared to an increase in the control group. However, a contradictory report was found in another study [57], which reported improvements in emotional exhaustion and personal accomplishment. IBSR intervention also includes weekly group meetings (3.5 h per meeting) requiring teachers to set time aside. This contradiction indicates that more advanced research with a representative sample is needed to validate the impact of IBSR.

Another unique intervention is group sandplay [58], which has not been extensively researched among educators or other professionals. Sandplay has, however, been adopted among college students to improve their interpersonal sensitivity level, which was reported to be significantly lower than that before the intervention (*p* < 0.01) [81]. This intervention helped teachers’ overall stress-coping abilities by shifting from passive to active coping [58]. However, there was no direct measurement of changes in stress or burnout levels among the teachers and therefore, the results need to be further consolidated in future research. Furthermore, the dynamics and mechanisms employed in this intervention have yet to be extensively investigated among teachers; hence future research may further explore this gap.

### 4.1. Implications for Policy and Practice and Future Research Directions

First, the scales used to measure stress and burnout scores in the various studies identified through this scoping review differed. However, the Perceived Stress Scale-10 and Maslach Burnout Inventory-Educator Survey (MBI-ES) were the most commonly used. The use of different scales makes an effective comparison of the efficacy of all the interventions difficult. Future systematic reviews and meta-analyses can target studies in which only specific valid and reliable scales were used for uniformity and accuracy when reporting and comparing the impact of these interventions on teacher stress and burnout.

Second, most of the interventions identified through this scoping review, although effective in mitigating teacher stress and burnout, require a considerable amount of time to complete, and this presents a barrier, as there is demand for teachers’ time is already significant. Considering the busy school environment and the issue of time for most of these interventions, alternative interventions, such as mobile text-based programs to reduce stress and burnout in teachers, can be explored. Mobile text technology is an evidence-based, innovative, convenient, easily accessible, low-cost, and scalable intervention. It has been adopted as a means of delivering psychological treatments and support for the public and patients [71,82,83,84,85]. Such innovations can easily be implemented at the school level to support teachers’ psychological needs. For example, the Wellness4Teachers program, currently being evaluated in Canada, is a CBT-based supportive messaging program that delivers daily text messages and mental health literacy information to teachers [85]. If found effective, this intervention can potentially reduce the time demand and the need for face-to-face interventions [55,58]. The outcomes of the Wellness4Teachers program evaluation will have implications for the support available to teachers to reduce their stress and burnout and improve their general well-being.

Third, this scoping review summarizes currently available interventions to address teacher stress and burnout. Although most identified interventions reported positive outcomes, the methodological quality of intervention studies has yet to be explored. Therefore, a meta-analysis or systematic review of these interventions is warranted to determine the levels of evidence of the intervention’s effectiveness.

Fourth, burnout has been described as an occupational illness, yet none of the interventions primarily focuses on causes such as overwork, lack of systemic support, work modifications, or specific work skills development. Future studies regarding interventions that prioritize and incorporate some of these parameters are needed.

Five, recently, Acceptance and Commitment Therapy (ACT), a ‘third wave’ more cognitive therapy updated version of CBT) has also been applied as an intervention for stress-related illnesses such as anxiety and depression [86]. Burnout has usually been linked with personal values related to cynicism [87]. Thus, ACT may be a better therapy than CBT or REBT, as it includes mindfulness and cognitive strategies and focuses on self-concept, values, psychological flexibility, and committed action to make a positive change, such as coping through efficacy.

Finally, given the physical stress responses inherent in burnout and the effectiveness of Progressive muscle relaxation (PMR) and autogenic training, it would be beneficial in the future to investigate other interventions that directly target the stress response, including activating the parasympathetic nervous system.

### 4.2. Limitations

The scoping review has some limitations. First, the search strategy was limited by the year of publication which may have excluded other potentially effective interventions. Second, only English language databases were searched; thus, some relevant studies in other languages may have been left out, impacting the interpretation of the findings. Third, the overall search strategy may have been biased towards health and sciences databases, and searching other bibliographic databases may have generated additional relevant studies. Five, the sample size for most of the individual studies included in this scoping review was small. Finally, two of the included studies, Okeke et al. 2021 [47] and Obiweluozo et al. 2021 [45] were published in the same year, utilizing the exact same study sample, sample size, study setting and intervention. The two studies also had the same study objectives, methods, flow chart and results, even though they had different authors and were published in different journals. Despite the similarities between the two manuscripts, the different authors made it impossible to determine which of the two manuscripts to exclude from the review. Notwithstanding these limitations, this scoping review offers good insight into the interventions used to reduce stress and burnout among teachers.

## 5. Conclusions

Stress and burnout in the teaching profession are widespread, and intervention to reduce these problems warrants attention both at the level of policy and practice. This review summarizes and discusses interventions that have been used to mitigate educator stress and burnout. This summary of the evidence may help inform health and education leaders to develop policies and adopt programs that are effective in addressing teacher stress and burnout. The review identified several effective interventions to address stress and burnout, although there are some shortfalls, especially regarding time constraints. Due to teachers’ busy schedules, time-consuming interventions may be challenging to undertake or may even add to teachers’ stress. Notwithstanding, schools need to promote and prioritize some of these interventions specifically designed to reduce teachers’ stress and burnout. Implementing suitable school-based interventions at all schools is appropriate and necessary to improve teachers’ stress-coping ability with the expectation that this will prevent or reduce the likelihood of burnout. Future studies need to investigate the effectiveness of teacher stress and burnout reduction programs that do not have time constraints and that are cost-effective, geographic location independent and easily scalable, such as the Wellness4Teachers program in Canada. The virtual nature of such programs may offset the need for teachers’ physical presence at a particular set time. Despite both the methodological differences and variations in the interventions used in the studies included in this scoping review, each of the interventions was found to reduce educator stress and burnout. These methodological differences and the quality of the included studies make it impossible to draw conclusions about which interventions are most effective in supporting educators’ mental health based on this scoping review.

Researchers need to work in partnership with governments, policymakers and school boards in the design, co-creation, implementation, monitoring and evaluation of teacher wellness initiatives to ensure their adoption.

## Figures and Tables

**Table 2:** Rational-Emotive Therapy Intervention.

Authors/Year	Country (Continent)	Study Design	Intervention: Focus and Content	Study Procedures	Participants, Sample Size	Age (Range, Mean, SD)	Scales Used to Measure Primary Outcome	Key Findings
Nwabuko et al., 2020 [44]	Nigeria (Africa)	RCT	Focus: Burnout Content: 32 group therapeutic sessions of Rational-Emotive Adult Education intervention (REAEI), organized over 16 weeks (two sessions per week). Four follow-up sessions over two weeks, three months after the last session	Teachers were randomized to treatment or CG.	Primary school teachers n = 86	NR	The Teacher Burnout Inventory (TBI)	TBI scores were lower in the treatment group compared with the CG both after therapy and at the three- month follow up (*p* < 0.0001). At follow-up assessment, there was a further reduction in burnout symptoms in the treatment group compared with participants in the CG (*p* < 0.0001).
Obiweluozo et al., 2021 [45]	Nigeria (Africa)	Group-RCT (waitlist)	Focus: Stress Content: Blended “Rational Emotive Behavior Therapy” (bREBT).12-weekly sessions, 2 h face-to-face session, followed by 12 weeks online REBT sessions.	Teachers were randomly assigned to the bREBT group, or WLC group. Multiple assessments.	Special education teachers n = 83	bREBT: mean age = 31.0 years WLG: mean age = 33.3	Teachers Stress Inventory (TSI). Single Item Stress Questionnaire (SISQ),	Mean perceived stress, stress symptoms, and total teachers’ stress score of the bREBT group at post-test and follow-up reduced significantly, compared to the WLC group. bREBT group mean stress manifestation (SM) reduced significantly, (*p* = 0.000), compared to WLG during Time 2.
Ogba et al., 2020 [46]	Nigeria (Africa)	RCT (waitlist)	Focus: Stress Content: Rational Emotive Occupational Health Coaching (REOHC). 12 weeks session 2- hour per week.	Teachers were randomly assigned to an immediate IG(IG) or WLC group After the pretest exercise. Post- and follow-up evaluations were conducted respectively at 2 weeks and 3 months after the REOHC program.	Special education teachers (autism) n = 87	IG: mean age = 31.0 years WLG: mean age 33.3 years.	Perceived Occupational Stress Scale and Stress Symptom Scale; Single Item Stress Questionnaire (SISQ).	The perceived stress and stress symptoms of the IG reduced significantly compared to WLG participants at post-test, and follow-up assessments.
Okeke et al., 2021 [47]	Nigeria (Africa)	Group-RCT (waitlist)	Focus: Stress Content: Blended Rational Emotive Occupational Health Coaching. (bREOHC). 12 weeks session, 2 h a day weekly intersession face-to-face session. Each session was followed by a practice exercise. Then 12 weeks online REBT sessions.	Participants were assigned to bREOHC group or CG.	Special education teachers n = 83	NR	Single Item Stress Questionnaire (SISQ), Teachers Stress Inventory (TSI).	Perceived stress, stress symptoms and the total teachers stress scores of the bREOHC group at post-test and follow up reduced significantly, compared to the WLC group.
Onuigbo et al., 2018 [48]	Nigeria (Africa)	Group-RCT	Focus: Stress Content: Rational Emotive Behavior Therapy (REBT). 12 weeks of 1.5 h sessions each and a 2-week follow-up program for.	Participants were randomly assigned to either a treatment group (n = 43) or no-intervention CG (n = 43).	Special education teachers n = 86	Treatment Group: mean age = 39.4 years (SD = 7.99) IG: mean age 39.4 (SD = 8.0)	Teachers’ Stress questionnaire (TSQ)	The REBT group experienced a significant mean decline in stress levels both at post-treatment and follow-up (*p* < 0.001) The CG showed no improvements at either post-treatment or follow-up sessions.
Ugwoke et al., 2018 [49]	Nigeria (Africa)	RCT	Focus: Burnout Content: 12-week Rational-Emotive Stress Management (RESM) intervention program; weekly 2 h sessions.	Participants were allocated to either the treatment group (n = 28) or the WLC group (n = 26). Multiple assessments.	Special education teachers n = 54	Mean age = 36.7 years	Teacher Burnout Scale (TBS) from the Shirom-Melamed Burnout Questionnaire	Significant decrease in EE among treatment group compared to the WLC group (*p* = 0.000). Significant decrease in job burnout (*p* = 0.000).
Uzodinma et al., 2022 [50]	Nigeria (Africa)	Group-RCT waitlist design	Focus: Burnout Content: Rational Emotive Occupational Health Coaching (REOHC). Weekly for 12 weeks. 2-h sessions Each session was followed by practice exercises.	All participants were randomly allocated to REOHC (n = 43) or WLC group (n = 43). Multiple assessments.	Special education teachers n = 86	Mean age = 32.3 years	Maslach Burnout Inventory—Educators Survey Educators (MBI-ES)	Significant decrease in burnout in REOHC in IG. (*p* = 0.000) compared to WLG at Time 2. Time 3 evaluation and follow-up 2 (Time 4), (*p* = 0.000).

TSI—Teacher stress inventory, DP—Depersonalization, PA—Personal accomplishment, EE—Emotional exhaustion, Y-CBT—Yoga-Based Cognitive Behavioral Therapy, CBT—Cognitive Behavioral Therapy, MBI—Mindfulness-Based Intervention, MBSR—Mindfulness-Based Stress Reduction, WLC—Waitlist control, RCT—Randomized control trial, NR—Not reported, IG—Intervention group, CG—Control group, CASS—Classroom Assessment Scoring System, SD—Standard Deviation, h (s)—Hour (s).

**Table 3:** Other interventions to reduce stress and burnout in teachers.

Authors/Year	Country	Study Design	Intervention: Focus and Content	Study Procedures	Participants, Sample Size	Age (Range, Mean, SD)	Scales Used to Measure Primary Outcome	Key Findings
Chesak et al., 2019 [51]	USA (North America)	Non RCT: Prospective, single-group follow-up study	Focus: Stress Content: Stress Management and Resiliency Training Program (SMART). Initial 1.5 h session for discussion of the essential components of the program. The online program consisted of 12 self-paced modules. Weekly emails were sent to the participants (weeks 8 through 52) of the study. Eight 1-h teleconferences were provided at regular intervals.	SMART program participants completed a follow-up survey at 2, 6, and 12 months.	Teachers n = 55	Mean age = 47.7 (SD = 9.9)	Perceived Stress Scale (PSS-10)	Stress was significantly lower at each follow-up (2, 6, and 12 months) compared with baseline (*p* = 0.003).
Chirico et al., 2020 [52]	Italy (Europe)	RCT	Focus: Burnout Content: Christian prayer and prayer-reflection. Combination of individual Christian prayer and a focus group of prayer-reflection. Participants received two 30 min training sessions a week over 2 months.	Teachers were randomized into two: prayer treatment (n = 25) or CG (n = 25).	Teachers n = 50	IG: mean age = 35.6 years (SD = 6.8) CG: mean age = 37.5 years (SD = 8.2).	Maslach Burnout Inventory- Educators Survey (MBI-ES), Italian version	Significant improvement across all outcome measures in the treatment group; EE (*p* < 0.001), DP (*p* < 0.001) with moderate to large effect size.
Dewi et al., 2018 [53]	Indonesia (Asia)	Non RCT: Quasi-experimental design	Focus: stress Content: Daily Progressive Muscle Relaxation with Music and Aromatherapy (PMR). Four sessions in four days. Each session lasted for 20 min. During the intervention, music was also played for 20 min.	Teachers were evenly assigned to the IG and CG.	Vocational and high school teachers n = 46	The in the IG: mean age = 32.4 years CG: mean age = 32.8 years	Teacher Stress Inventory (TSI)	Stress level in the IG decreased compared to participants in CG (*p* = 0.000), indicating significant differences in the stress levels between the IG and the CG.
Schoeps et al., 2019 [54]	Spain (Europe)	Non RCT: Quasi-experimental design	Focus: Stress, burnout Content: The Ability Model of Emotional Intelligence Training Program. Five 2 h sessions, over three months in groups of 15–20 teachers.	Participants were assigned to the IG (n = 135) or to the CG (n = 205). Outcome measures were collected before the training (T1), after the training (T2), and at 6--month follow-up (T3).	Teachers n = 340	Age range 22 to 63 years Mean age = 42.6 years (SD = 9.00).	Spanish Burnout Inventory. Depression, Anxiety and Stress Scales (DASS-21), Spanish version	IG showed marginal significant differences for burnout (*p* = 0.06), but no significant differences for emotional symptoms, (*p* = 0.31). Reduced levels of work-related stress and emotional symptoms in IG compared to the CG.
Kim & Gurvitch, 2020 [55]	USA (North America)	Non RCT: Mixed-methods, quasi-experimental design	Focus: Stress Content: Sports-Based Physical Activity Program included 7 weeks of training in Catchball, a team sport and a tournament as the culminating event.	Program participants IG (n = 12) or non-program participants CG (n = 20).	Teachers n = 32	IG: age range 24 to 55 Mean age = 40.1 years CG: age range 28 to 67 Mean age = 47.5 years.	Perceived Stress Scale (PSS-10)	Qualitative result: Sports-Based Physical Activity Program helped decrease the teachers’ stress level. PSS score did not appreciably change.
Oliveira et al., 2022 [56]	Portugal (Europe)	Non RCT: Quasi-experimental study	Focus: Stress, burnout Content: A+ intervention, an online social and emotional learning intervention.	42 assigned to the experimental group. Data on the efficacy of the A+ was collected across four waves using a set of self-report questionnaires	Elementary-school teachers n = 81	Mean age = 46.2 years (SD = 4.8),	Perceived occupational stress scale. Maslach Burnout Inventory—Educators Survey (MSB-ES) (Portuguese version)	A+ increased emotional wellbeing, decreased occupational stress and EE symptoms.
Schnaider-Levi et al., 2020 [57]	Israel (Asia)	Non RCT: Quasi-experimental design.	Focus: Stress, burnout Content: Inquiry-Based Stress Reduction (IBSR) cognitive-reframing program.12-week IBSR program with 4.5 h of weekly engagement. The IBSR intervention included weekly group meetings (3.5 h per meeting) and weekly individual sessions with a facilitator (1 h/session) for 12 weeks	Prospective intervention with a passive CG.	Teachers n = 53	IG: mean age 46.3 years (SD = 6.5) CG: mean age 46.5 years (SD = 6.1)	Maslach Burnout Inventory -MBI Perceived Stress Scale (PSS-10); Depression, Anxiety, Stress Scales (DASS-21)	Teachers in the IG showed improvements in EE; (*p* = 0.01) and PA (*p* = 0.04). Significant changes in DASS scales were not observed within or between groups.
Sun et al., 2018 [58]	China (Asia)	Non RCT: Prospective controlled longitudinal study	Focus: Stress Content: Group sandplay. 3-h tutorial on the effect of psychological pressure at work and participants were introduced to the working principles and process of sandplay.	Participants were equally divided into two groups: An experimental sandplay group EG (n = 97) or CG (n = 97).	194 Teachers	EG (Mean age 36.9 years SD 12.2) CG (Mean age 37.5 years SD 10.6)	Simplified Coping Style Questionnaire	EG participants shifted from passive coping to active coping. Group sandplay effectively improved teachers’ overall stress coping abilities.
Thephilah et al., 2020 [59]	India (Asia)	Non RCT: Group comparison study	Focus: Stress, burnout Content: Autogenic relaxation Stress Management Program. 6 sessions, once a week for 6 weeks. Progressive steps are introduced and practiced for 15–20 min whilst concentrating on breathing and relaxation of muscles.	Teachers assigned to a CG (n = 14) and experimental group (n = 14).	Private and fully aided schoolteachers n = 28	Age range 25 to 60 years.	Maslach Burnout inventory teacher Stress Inventory (TSI)	Median value in the pre-test scores were significantly different from the median post-test scores for EE (*p* = 0.02). Work-Related Stressors (t = 244 *p* = 0.05) subscales
Zadok-Gurman et al., 2021 [60]	Israel (Asia)	Non RCT: Prospective controlled trial	Focus: Stress, Burnout Content: Blended Inquiry-Based Stress Reduction (IBSR). 10 biweekly group meetings (2.5 h/meeting) and biweekly individual sessions with a facilitator (1 h/session) for 20 weeks. Face-to-face, and online learning.	IBSR IG (n = 35) and CG (n = 32). The sessions were conducted in blended learning that included traditional learning (face-to-face) and online learning.	Teachers n = 67	Age ranged between 34 to 67 (M = 45) years	Perceived Stress Scale (PSS-10). Maslach Burnout Inventory (MBI)	Teachers in both IG and CGs showed deterioration in EE scores but IG was less substantial than in the CG (*p* < 0.01). PSS-10 scores: no differences were observed between the groups.

TSI—Teacher stress inventory, DP—Depersonalization, PA—Personal accomplishment, EE—Emotional exhaustion, Y-CBT—Yoga-Based Cognitive Behavioral Therapy, CBT—Cognitive Behavioral Therapy, MBI—Mindfulness-Based Intervention, MBSR—Mindfulness-Based Stress Reduction, WLC—Waitlist control, RCT—Randomized control trial, NR—Not reported, IG—Intervention group, CG—Control group, CASS—Classroom Assessment Scoring System, SD—Standard Deviation, h (s)—Hour (s).

## Data Availability

Not applicable.
